# Aversive Counterconditioning Attenuates Reward Signaling in the Ventral Striatum

**DOI:** 10.3389/fnhum.2016.00418

**Published:** 2016-08-19

**Authors:** Anne Marije Kaag, Renée S. Schluter, Peter Karel, Judith Homberg, Wim van den Brink, Liesbeth Reneman, Guido A. van Wingen

**Affiliations:** ^1^Department of Radiology, Academic Medical CenterAmsterdam, Netherlands; ^2^Department of Psychiatry, Academic Medical CenterAmsterdam, Netherlands; ^3^Amsterdam Brain and Cognition, University of AmsterdamAmsterdam, Netherlands; ^4^Donders Institute for Brain, Cognition, and Behaviour, Medical Centre, Radboud UniversityNijmegen, Netherlands; ^5^Spinoza Center for Neuroimaging, Institute of the Royal Netherlands Academy of Arts and SciencesAmsterdam, Netherlands

**Keywords:** ventral striatum, reward reinstatement, fMRI, counterconditioning

## Abstract

Appetitive conditioning refers to the process of learning cue-reward associations and is mediated by the mesocorticolimbic system. Appetitive conditioned responses are difficult to extinguish, especially for highly salient reward such as food and drugs. We investigate whether aversive counterconditioning can alter reward reinstatement in the ventral striatum in healthy volunteers using functional magnetic resonance imaging (fMRI). In the initial conditioning phase, two different stimuli were reinforced with a monetary reward. In the subsequent counterconditioning phase, one of these stimuli was paired with an aversive shock to the wrist. In the following extinction phase, none of the stimuli were reinforced. In the final reinstatement phase, reward was reinstated by informing the participants that the monetary gain could be doubled. Our fMRI data revealed that reward signaling in the ventral striatum and ventral tegmental area following reinstatement was smaller for the stimulus that was counterconditioned with an electrical shock, compared to the non-counterconditioned stimulus. A functional connectivity analysis showed that aversive counterconditioning strengthened striatal connectivity with the hippocampus and insula. These results suggest that reward signaling in the ventral striatum can be attenuated through aversive counterconditioning, possibly by concurrent retrieval of the aversive association through enhanced connectivity with hippocampus and insula.

## Introduction

Appetitive conditioning is the process by which cues (CS) become associated with reward (US) and subsequently acquire incentive salience (reward motivation) themselves CR (Everitt and Robbins, 2005). Once these CRs are acquired they are difficult to extinguish, especially for highly salient reward such as food and drugs (Myers and Carlezon, 2010). For example, even after an extended period of abstinence, cue-induced reinstatement can occur after a single re-exposure to the drug itself, an associated stimulus or, environmental stress (Shaham et al., 2003). It is therefore important to gain insight into the mechanisms that underlie reward reinstatement and to find procedures to prevent reinstatement.

One of the possibilities to prevent reward reinstatement could be counterconditioning. This is the process of pairing of a CS+ with an US that has a valence opposite to the valence of the original US (e.g., unpleasant shock experience versus pleasant drug experience; Pearce and Dickinson, 1975). Several studies have shown that counterconditioning can reduce fear CRs (e.g., Paunović, 2003; Raes and De Raedt, 2012) and appetitive sexual responses (e.g., Tanner, 1974). However, studies that used counterconditioning to change appetitive CRs are scarce. An early, non-experimental study suggested that the administration of electrical shocks to addicted individuals while they relived past drug using experiences can result in a low relapse rate during the 2-years follow-up (Copeman, 1976). Despite this early positive finding of aversive counterconditioning on relapse in drug addiction, similar effects have only recently been reported in experimental studies in which it was demonstrated that aversive counterconditioning is more effective than extinction in reducing reward motivation in humans (Van Gucht et al., 2010) and rats (Tunstall et al., 2012). However, two important questions remain to be answered: (1) Does aversive counterconditioning also modulate reward reinstatement, and (2) what are the neurobiological mechanisms involved in aversive counterconditioning? This knowledge may help to develop aversive counterconditioning strategies to prevent relapse in addiction.

The mesocorticolimbic pathway, which connects the ventral tegmental area to the striatum, amygdala, hippocampus, ventral- and dorsal medial prefrontal cortex plays an essential role in both reward and fear learning (Everitt and Robbins, 2005; Abraham et al., 2014). A recent animal study demonstrated that the ability to learn the association between a cue and a negative outcome was retarded if that stimulus was previously paired with a rewarding outcome. This effect was associated with heightened neural activity within the thalamus, insula, amygdala, and ventral striatum (Nasser and McNally, 2012). To our knowledge, there is currently only one human study that reported on the neural correlates of counterconditioning, using a paradigm in which stimuli that previously predicted an aversive outcome, were paired with a rewarding outcome. Using this paradigm it was demonstrated that counterconditioning of conditioned fear responses was associated with reduced amygdala and hippocampus activation following reinstatement (Bulganin et al., 2014). Previous studies demonstrated that the prospect of pain reduces reward signaling within the ventral striatum (Talmi et al., 2009), and that the processing of positive and negative reward interact on a neural level (Park et al., 2011). However, there are no functional magnetic resonance imaging (fMRI) studies that assessed the neural correlates of aversive counterconditioning aiming to reduce (the reinstatement of) reward CRs. While based on these previous study it could be expected that aversive stimuli can be used to alter appetitive CRs, there are several indications that appetitive CRs are more difficult to alter than aversive CRs: for instance, while exposure treatments for anxiety disorders are highly successful in the extinction of fear CRs, the same exposure treatments are fairly unsuccessful in reducing drug-CRs and thus the treatment of substance use disorder (Kaplan et al., 2011). The current study therefore aims to investigate the neural mechanisms related to aversive counterconditioning of conditioned reward responses, specifically focussing on the effects of counterconditioning on reward reinstatement.

Given the increasing evidence of mesocorticolimbic interactions between aversive and appetitive learning, we hypothesized that aversive counterconditioning would reduce reward signaling following reinstatement within the mesocorticolimbic pathway. To test this hypothesis we developed an fMRI task that consisted of four phases: first, in the conditioning phase, two different stimuli were reinforced with a monetary reward if the participant hit the target on time. Second, in the counterconditioning phase, one of the stimuli was paired with an aversive electrical shock to the wrist. Third, in the extinction phase, none of the stimuli were reinforced neither by reward nor by shock. Fourth, in the reinstatement phase, reward was reinstated by informing the participants that the monetary gain could be doubled. It was expected that reward reinstatement in the ventral striatum would be prevented only for stimuli that were counterconditioned, but not for stimuli that were not counterconditioned. Because the ventral striatum is connected with several cortical and limbic regions, we also applied a functional connectivity analysis with the ventral striatum as seed region, as this may further clarify the underlying mechanisms involved in counterconditioning.

## Materials and Methods

### Participants

Twenty-five healthy volunteers participated in this study (mean age 22.0 ± 2.1 SD, 12 women). Participants were recruited at the University of Amsterdam. All participants had normal vision and reported taking no medication affecting the nervous system, including no illicit drugs. None of the participants used nicotine or more than 21 units of alcohol per week. Participants were reimbursed for their time (€15) and won an additional €10 in the counterconditioning task. The study was approved by the ethics committee of the University of Amsterdam and all participants gave written consent to participate in the study.

### Experimental Paradigm

**FIGURE 1:**
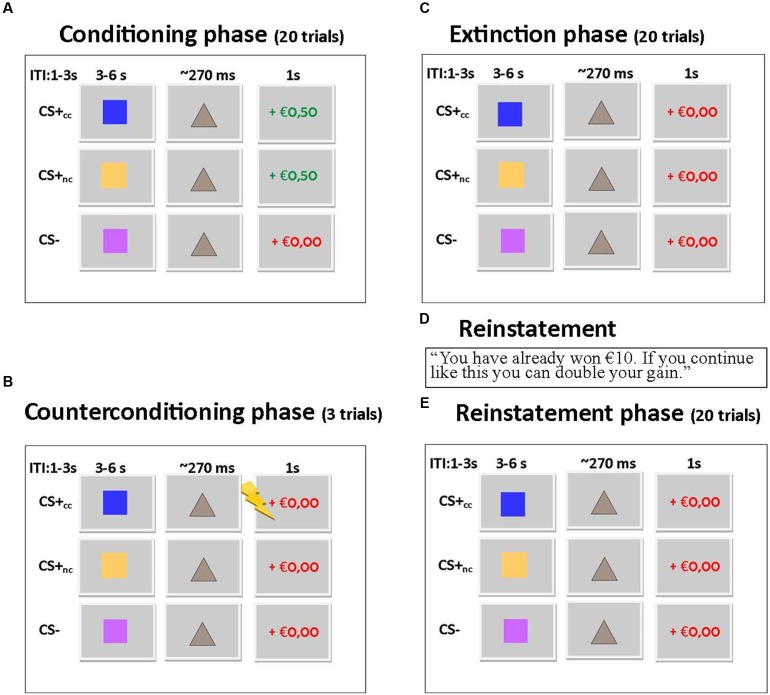
**(A)** Trial sequence for conditioning phase. 50% of the CS+ trials were reinforced with a monetary gain. Each stimulus is presented 20 times. **(B)** Trial sequence for counterconditioning phase. The CS+_cc_ stimuli are reinforced with an electrical shock, the other stimuli are not reinforced. Each stimulus is presented three times. **(C)** Trial sequence for extinction phase. None of the stimuli is reinforced. Each stimulus is presented 20 times. **(D)** Reward is reinstated by showing this text. **(E)** The trial sequence for the reinstatement phase is similar to the trial sequence of the extinction phase. Each stimulus is presented 20 times.

The counterconditioning task was based on the monetary incentive delay task (Knutson et al., 2000; Ossewaarde et al., 2011; Bulganin et al., 2014) and consisted of four phases: a conditioning phase, a counterconditioning phase, an extinction phase and a reinstatement phase (see **Figure 1**). During each phase the same stimuli (blue, yellow, and purple squares; colors were counterbalanced across subjects) were shown: the counterconditioned CS+_cc_ predicted a monetary reward during the conditioning phase but an aversive electrical shock to the wrist during the counterconditioning phase and was unreinforced during the other phases. The non-counterconditioned CS+_nc_, predicted a monetary reward during the conditioning phase, but was unreinforced during the other phases. The CS- was never reinforced. The monetary reward consisted of an image of €0,50. Participants were instructed that they could win an amount up to €10. The intensity of the electrical shock was individually adjusted, to be unpleasant but not painful. In the conditioning, extinction, and reinstatement phase each stimulus was presented 20 times. In the counterconditioning phase, each stimulus was presented three times. Across all phases, each trial started with a cue (the CS+_cc_, the CS+_nc_, or the CS-) presented for 3000–6000 ms, followed by a target. If subjects responded to the target on time, the trial was reinforced with a monetary gain. The initial target duration was 270 ms. With every hit, the target duration was reduced by 18 ms, with every miss the target duration was increased by 36 ms. In this way the target duration was manipulated to ensure that approximately 50% of the trials were ‘hit’ trials and thus reinforced resulting in 10 reinforced CS+_cc_ and 10 reinforced CS+_nc_ trials. Thus, the CS was reinforced in 50% of the trials. As a result, subjects would always gain a total of €10. Although, the CS- trials are never reinforced, the target duration in these trials is manipulated in the same way as the target duration for the CS+ trials. Feedback followed target offset immediately, and was displayed for 1000 ms The inter-stimulus interval varied between 1000 and 3000 ms. Before task onset, subjects were instructed which stimuli predicted a reward but that they had to respond to the target in all trials (including the non-rewarding trials). Doing so ensures that differences in neural responsiveness cannot be due to differences in motor preparation. Also, subjects were informed that they could receive an electrical shock, but were not instructed about contingency. Normally, in fear conditioning studies, fear is reinstated by simply presenting the US (the electrical shock) without the CS (Bulganin et al., 2014). In the current study, however, the US was the visual presentation of €0,50, which they would later be received. We assumed that simply displaying the visual stimulus of €0,50 to reinstate reward conditioning, which would have been in line with previous fear conditioning studies, would not be sufficient to reinstate reward anticipation. Therefore, we aimed to reinstate the reward CR by presenting the following text to the participants: “*You have already won €10. If you continue like this and keep responding on time, you can double your gain*.” However, none of the post-reinstatement trials were actually reinforced. After the task, CS–US contingency awareness and CS valence were assessed using a visual analog scale.

This experimental paradigm was developed to study the effects of counterconditioning on reward reinstatement, but we wanted to prevent the relationship between the CS+cc and the shock to be too strong. Therefore the counterconditioning phase had a limited number of trials and as a consequence this phase could not be included in the fMRI and behavioral analysis.

### Valence and Contingency Ratings

Contingency awareness and valence for each CS was assessed using a visual analog scale ranging from 0 to 10: “How pleasant/unpleasant did you find this square?” and “How likely was it that this square was followed by a shock.” Subjects were classified as being unaware of the stimulus-shock contingency if they indicated that the change of getting a shock after seeing the CS- was larger than zero and/or when they indicated that the change of getting a shock after seeing either CS+ stimulus was equal to zero.

### Functional Magnetic Resonance Imaging Data Acquisition and Analysis

Images were acquired on a 3.0-T Achieva full-body scanner (Philips Medical Systems, Best, the Netherlands) using a 32 channel SENSE head coil. Echo planar images (EPIs) were taken covering the whole brain, with a total of 37 ascending axial slices (3 mm × 3 mm × 3 mm voxel size; slice gap 3 mm; TR/TE 2000 ms/28 ms; matrix 80 × 80). A T1-3D high resolution anatomical scan (TR/TE 8.2/3.7; matrix 240 × 187; 1 × 1 × 1 voxel; transverse slices) was acquired for spatial normalization purposes. Imaging data were analyzed using SPM8^1^. Preprocessing included realignment, slice-time correction, co-registration of the structural and functional scans, normalization to MNI-space based on the segmented structural scan and smoothing with a kernel of 8 mm full-width at half maximum. First-level models included separate regressors for the CS+_cc_, CS+_nc_ and CS-, which were modeled separately for the four different phases. These regressors were convolved with the canonical hemodynamic response function with a duration of 0 s. Six realignment parameters were included as regressors of no interest. A high pass filter (1/128 Hz) was included in the first level model to correct for low frequency signal drift. The contrast images were entered into a second-level full-factorial design.

To assess whether the paradigm resulted in a significant reward CR, we tested the main effect of stimulus (CS+_cc_ and CS+_nc_ versus CS-) during the conditioning phase. Subsequently we tested the effect of counterconditioning on extinction and reinstatement by testing the main effect of stimulus type (CS+_cc_ versus CS+_nc_) during extinction and reinstatement. To assess whether counterconditioning resulted in a reduction of reward signaling, we tested a phase (conditioning versus reinstatement) by stimulus type (CS+_cc_ versus CS+_nc_) interaction effect.

In order to assess whether differences in ventral striatal responsiveness were also associated with differences in ventral striatal functional connectivity, we used generalized psycho-physiological interaction analysis (gPPI; McLaren et al., 2012) with the ventral striatum as a seed region to compare functional connectivity between the counterconditioned and non-counterconditioned CS+ conditions after reinstatement. This type of analysis allows to investigate changes in functional connectivity with a seed region (in this case the ventral striatum) related to a certain psychological variable (in this case the presentation of either a counterconditioned or non-counterconditioned stimulus). The time series of the first eigenvariate of the BOLD signal were temporally filtered, mean corrected, and deconvolved to generate the time series of the neuronal signal for the ventral striatum (which was defined as the nucleus accumbens from the Harvard–Oxford subcortical structure probability atlas) for each individual subject. The interaction term – PPI – was computed by multiplying the time series from the psychological regressors with this physiological variable.

All voxel-wise statistical tests are family wise error rate corrected for multiple comparisons (*p* < 0.05). Whole brain analysis were corrected on the cluster level, using an initial height threshold on voxel level of *p* < 0.01. Because of the *a priori* hypothesis on the role of the ventral striatum in appetitive conditioning, small-volume corrections (SVCs) were applied using the same ventral striatal mask that was used in the PPI analyses (Worsley et al., 1996). The anatomical names of all significant clusters were identified using the Automatic Anatomated Labeling atlas toolbox in SPM8 (Tzourio-Mazoyer et al., 2002). To explore a possible correlation between brain activation and behavior, the individual change in valence ratings of the CS stimuli were entered as a covariate in the whole brain analyses.

### Behavioral Data Analysis

The effects of counterconditioning on reaction time was assessed in a repeated measurements (rm) ANOVA with a test for a stimulus (CS+_cc_, CS+_nc_, CS-) by phase (conditioning, extinction, reinstatement) interaction effect using SPSSv20 (Statistical Package for the Social Sciences). A one way ANOVA was used to test for differences in valence ratings for each CS type.

## Results

### Contingency and Valence Ratings

Nine participants (34.6%) were unaware of the contingency between the stimulus and the electrical shock. As these participants did not learn the association between the cue that predicted the electrical shock and the electrical shock itself, they were excluded from further analysis. The final sample therefore consisted of 16 participants (mean age 22.1 ± 2.0 SD, nine women). The rmANOVA showed that there was a significant difference in CS-valence ratings (*F*_2,30_ = 4.0, *p* = 0.03). Follow-up paired sampled *t*-test indicated that there was no significant difference in valence rating between the CS+_cc_ and CS-, whereas, the valence of CS+_nc_ was rated significantly higher than the valence of the CS+_cc_ (*t*_15_ = 3.1, *p* = 0.007) and CS- (*t*_15_ = 2.6, *p* = 0.02). In other words, these results indicate that subjects ‘liked’ the stimulus that predicted a monetary gain in the conditioning phase better than the stimulus that was additionally counterconditioned with aversive shocks.

### Reaction Times

Repeated measurements ANOVA’s revealed no stimulus by task phase interaction effect (*F*_6,10_ = 0.41, *p* = 0.86) and no main effect of task phase (*F*_3,13_ = 1.34, *p* = 0.31). However, there was a main effect of stimulus type (*F*_2,14_ = 19.67, *p* < 0.001) across all phases,. Follow-up tests revealed that there were no significant differences between the CS+_cc_ and CS+_nc_ reaction times (*F*_1,15_ = 1.73, *p* = 0.29). However, the reaction times to the CS- were significantly longer than the reaction times the other two stimuli (*F*_1,15_ = 40.72, *p* < 0.001): CS- = 280.51 (mean) ± 7.91 (standard error), CS+_cc_ = 262.22 ± 8.72 and CS+_nc_ = 267.22 ± 8.72. These results indicate that counterconditioning did not significantly affect the reaction time to the reward predicting cues.

### Functional Magnetic Resonance Imaging

All results described below are family wise error rate corrected for multiple comparisons (*p* < 0.05). The exact statistics can be found in **Table 1**.

**Table 1:** Main effects of appetitive conditioning and effect of aversive counterconditioning on reward-reinstatement.

	Cluster	Cluster	Peak voxel	Peak voxel			Voxel region
					
	# voxels	*p*-value	*z*-value	MNI-coordinates		
**Conditioning**	3220	<0.001	4.34	-2	-6	62	L superior motor area
CS+ > CS-			3.77	-34	-20	52	L precentral
			3.58	-46	-12	56	L post-central
			3.55	-38	-26	54	L post-central
			3.39	-6	0	44	L middle cingulate
	1424	<0.001	4.18	6	14	-4	R caudate
			3.43	-10	12	-4	L caudate
			3.07	6	-18	10	R thalamus
			2.69	-6	24	-6	L olfactory
	81	0.008	3.43	-10	12	-4	L nucleus accumbens^a^
	110	0.001	4.18	6	14	-4	R nucleus accumbens^a^
CS- > CS+							
	1642	<0.001	4.48	-42	14	42	L middle frontal gyrus
	1456	0.001	4.44	-44	-56	36	L angular gyrus
			4.4	-54	-56	40	L inferior parietal
	881	0.013	4.4	50	-56	24	R angular
	2285	<0.001	4.11	-18	40	36	L superior frontal
			3.85	16	26	54	R superior frontal
			3.72	-40	46	-8	L orbital middle fronal
			3.45	-10	28	52	L medial superior frontal
			3.34	-18	56	28	L superior frontal
			3.26	8	42	44	R medial superior frontal
			3.23	-44	42	-10	L orbital inferior frontal
			3.07	-8	32	50	L medial superior frontal
CS+nc > CS+cc	No significant voxels						
CS+cc > CS+nc	No significant voxels						
**Extinction**							
CS+cc > CS+nc	2191	<0.001	3.69	-14	-38	-20	L cerebellum
			2.8	12	-34	-20	R cerebellum
			2.53	-22	-44	-8	L lingual gyrus
**Reinstatement**							
CS+cc > CS+nc	No significant voxels						
CS+nc > CS+cc	3702	<0.001	4.65	24	-54	-50	R cerebellum
			3.82	-28	-58	-50	L cerebellum
	1072	0.004	3.72	60	-34	50	R supramarginal gyrus
			3.72	46	-30	50	R post-central gyrus
			3.72	42	-40	52	R inferior parietal
			3.45	34	-48	56	R superior parietal
	20	0.034	2.91	12	10	-6	R nucleus accumbens^a^


*These contrasts reflect the difference between the CS- and both CS+ stimuli (CS+cc and CS+nc). All results were *p* < 0.05, cluster level FWE corrected with an initial height threshold of *p* = 0.01 uncorrected. ^a^Corrected for the volume of the right nucleus accumbens, *p*_*peak voxel*_ < 0.05.*

During conditioning the CS+ (counterconditioned and non-counterconditioned) versus CS- contrast showed significant activation of the ventral striatum, caudate nucleus and bilateral motor cortex and a significant deactivation of the superior, middle, and inferior prefrontal cortex. These results indicate that the task indeed elicited significant activation of the reward network. In the conditioning phase there were no significant difference in neural activation between the counterconditioned and non-counterconditioned CS+.

**FIGURE 2:**
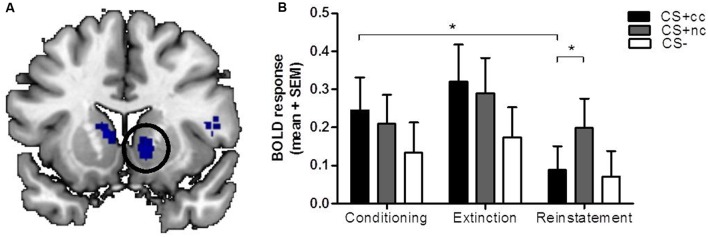
**The neural response to counterconditioned versus non-counterconditioned CS+.** The figures on the left **(A)** show the neural responses after reinstatement to the counterconditioned versus the non-counterconditioned CS+stimuli in the ventral striatum. The bar graphs on the right **(B)** are a visual presentation of the response to the counterconditioned CS+ (CS+_cc_; black), non-counterconditioned CS+ (CS+_nc_; gray) and CS- (white) at the peak voxel (MNI: 12 10 -6, *z* = 2.91). Compared to the conditioning phase, ventral striatal BOLD response during reinstatement is significantly decreased for the counterconditioned CS+, but not for the other stimuli. During reinstatement, the ventral striatal BOLD response for the non-counter CS is significantly higher compared to the BOLD response for the counterconditioned stimulus. The figures are displayed at *p* < 0.001 uncorrected for visualization purpose. ^∗^ = whole brain significant stimulus type (CS+_cc_ versus CS+_nc_) by phase (conditioning versus reinstatement) interaction effect.

During the extinction phase, the counterconditioned CS+ elicited more activation than the non-counterconditioned stimulus in the cerebellum and lingual gyrus, but not the ventral striatum. Importantly, during the reinstatement phase, however, the non-counterconditioned CS+ elicited more activation than the counterconditioned CS+ in the ventral striatum (small-volume corrected; **Figure 2A**) as well as in the bilateral cerebellum and regions within the parietal cortex. There was no significant interaction effect between stimulus type (CS+_cc_ and CS+_nc_) and phase (conditioning versus extinction). However, there was a significant interaction effect between stimulus type (CS+_cc_ and CS+_nc_) and phase (conditioning versus reinstatement) within the ventral striatum indicating that, compared to the conditioning phase, the ventral striatum response to the counterconditioned CS+ significantly reduced after reinstatement, whereas the ventral striatum response to the non-counterconditioned CS+ did not change (**Figure 2B**). These results suggest that aversive counterconditioning reduces reward signaling in ventral striatum following reinstatement. An exploratory regression analysis with the change in valence rating as covariate, did not reveal any significant brain-behavior correlations.

### Functional Connectivity

All results described below are were family wise error rate corrected for multiple comparisons (*p* < 0.05). The exact statistics can be found in **Table 2**.

**Table 2:** Differences in ventral striatal connectivity following reward reinstatement.

	Cluster size	Cluster	Peak voxel	Peak voxel			Voxel region
					
	*# voxels*	*p*-value	*z*-value	*MNI-coordinates*		
**Reinstatement**							
CS+cc > CS+nc	4186	<0.001	4.32	26	-6	-6	R pallidum
				12	-24	-12	Brainstem
				16	-4	12	R Thalamus
				16	-52	22	R precuneus
	4014	<0.001	4.18	16	-56	-46	R cerebellum
				-14	-56	-12	L cerebellum
				-30	-34	-4	L hippocampus
				-40	-20	20	L rolandic opperculum
				-36	-20	22	L insula
CS+nc > CS+cc	No significant voxels						


*All results were *p* < 0.05, cluster level FWE corrected with an initial height threshold of *p* = 0.01 uncorrected.*

**FIGURE 3:**
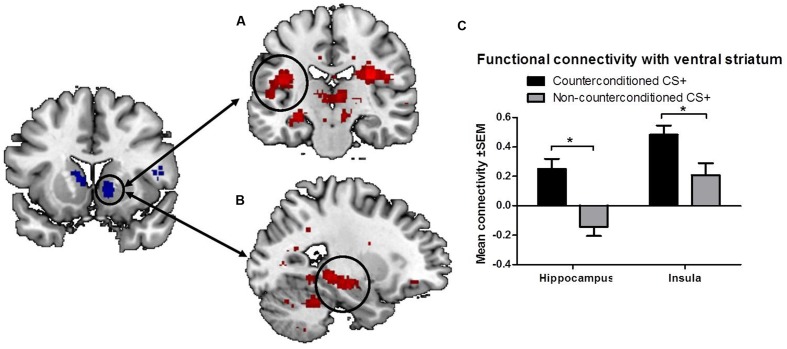
**Functional connectivity of the ventral striatum.** The figure shows significant differences in functional connectivity for the counterconditioned versus non-counterconditioned CS+ following reinstatement. Counterconditioning strengthened the functional connectivity of the ventral striatum seed region (left) with the left insula **(A)** and left hippocampus **(B)**. The figures are displayed at *p* < 0.001 uncorrected for visualization purpose. The graph **(C)** is a visual representation of the functional connectivity changes with the ventral striatum in response to counterconditioned and non-counterconditioned CS+, in the insula and hippocampus. ^∗^ = whole brain significant main effect of stimulus type (CS+_cc_ versus CS+_nc_) on ventral striatal connectivity following reward reinstatement.

To further explore the effects of aversive counterconditioning on functional connectivity of the ventral striatum after reinstatement, we conducted a psychophysiological interaction analysis with the right ventral striatum as seed region. This analysis compared functional connectivity of the ventral striatum following reinstatement during counterconditioned and non-counterconditioned CS+ conditions and showed that activity within the right ventral striatum following counterconditioned CS+ was more strongly coupled to activation in the left hippocampus, insula, and rolandic operculum, the right pallidum, thalamus and precuneus and the bilateral cerebellum than following non-counterconditioned CS+ (**Figure 3**; **Table 2**). Together with the fMRI analysis, these results indicate that counterconditioning increases functional connectivity to the ventral striatum, whereas it reduces neural responsiveness of the ventral striatum, following reward reinstatement.

## Discussion

The tendency of conditioned reward reinstatement, even after an extended period of abstinence, is one of the hallmarks of addiction (Shaham et al., 2003). The aim of this study was to gain a better insight in the neural mechanisms involved in conditioned reward reinstatement and to test whether aversive counterconditioning could attenuate the reinstatement of reward. The results demonstrate that aversive counterconditioning indeed reduced reward signaling within the ventral striatum following reward reinstatement. Dopaminergic projections from the ventral tegmental area to the ventral striatum and prefrontal cortex are considered to play a key role in the processing of reward and it is well-established that dopaminergic neurons within the ventral striatum increase their firing in response to CS that predict a reward (Schultz, 2007; Volman et al., 2013; Abraham et al., 2014). Thus, reduced reinstatement related BOLD response within the ventral striatum following aversive counterconditioning may reflect reduced dopaminergic firing and thus reduced reward anticipation/expectation. This is confirmed by the finding that, on a behavioral level, the valence of the counterconditioned stimuli was scored significantly lower compared to the non-counterconditioned stimuli. Thus previously rewarding stimuli that were paired with an aversive outcome were perceived as significantly less pleasant compared to previously rewarding stimuli that were never paired with an aversive outcome.

In addition to reduced ventral striatal responsiveness after reward reinstatement, we found that aversive counter-conditioning also strengthened ventral striatal connectivity with the insula, hippocampus, thalamus, rolandic operculum and cerebellum. The insula is a key region in interoceptive processing (Craig, 2009) and may therefore provide body-relevant information about the coding for aversiveness during reward related processing (Paulus and Stewart, 2014). For example, cues that predict an aversive outcome have been shown to elicit strong activation of the insula as well as the hippocampus, thalamus, rolandic operculum, and cerebellum (Sehlmeyer et al., 2009). The hippocampus is involved in the retrieval of aversive memory (Milad et al., 2007) and reward reinstatement (Fuchs et al., 2005; Rogers and See, 2007). Both the insula (Haber and Knutson, 2010; Sesack and Grace, 2010; Cauda et al., 2011) and the hippocampus (Baudonnat et al., 2013) are anatomically and functionally connected to the ventral striatum. Given this connection it has been suggested that these regions interact dynamically during reward processing and decision making (Baudonnat et al., 2013; Paulus and Stewart, 2014). Similarly, increased insula-hippocampus functional connectivity has been demonstrated during the consolidation of fear (Feng et al., 2013). Therefore, the current finding of increased striatal connectivity with the insula and hippocampus after aversive counterconditioning is likely to reflect enhanced retrieval of the aversive (bodily) state associated with the electrical shock, thereby disrupting the retrieval of the appetitive reward memory previously associated with the CS+, resulting in reduced reward signaling within the ventral striatum following reward reinstatement.

Previous studies have demonstrated that aversive counterconditioning is more effective than extinction in reducing appetitive CRs (e.g., cue-induced chocolate craving; Van Gucht et al., 2010) and several other studies observed an interaction between aversive and appetitive processes in associative-learning and decision making in the mesocorticolimbic pathway (Hu et al., 2013; Bulganin et al., 2014; Engelmann et al., 2015). Our results contribute to these previous findings by demonstrating that aversive counterconditioning attenuates reward signaling within the ventral striatum following reward reinstatement, possibly by enhancing striatal connectivity with the hippocampus and insula.

The results of our study also support the hypothesis that aversive counterconditioning could be a successful method for changing appetitive CRs in addiction (Copeman, 1976; Rossi et al., 1988; Van Gucht et al., 2010, 2013). However, addiction is generally characterized by compulsive drug seeking, defined as persisting in the face of adverse consequences (Vanderschuren and Everitt, 2004; Robbins et al., 2008). In line with this, addicted individuals typically show diminished behavioral and neural sensitivity to monetary punishment (Bechara and Damasio, 2002; Wrase et al., 2007; Bjork et al., 2008; Beck et al., 2009). This suggests that aversive counterconditioning may be an effective method to modify appetitive CRs in healthy individuals. However, even in healthy individuals it has been demonstrated that addicted patients may actually be insensitive to aversive counterconditioning. However, to our knowledge, this is the first fMRI study to report on findings of an experimental paradigm that enables the investigation of the effect of a negative consequence other than a monetary loss on reward anticipation. Using this paradigm to investigate aversive-appetitive interactions not only in healthy but also addicted individuals may thus allow for the development of better pharmacological or behavioral treatments for substance dependence (Abraham et al., 2014).

It should be noted, however, that while we aimed to extinguish the reward CR, the ventral striatum did not show a differential effect already during the extinction phase, immediately after aversive counterconditioning. Visual inspection of **Figure 2** may even suggest that ventral striatal activation during extinction was increased compared to the conditioning phase, although this effect was non-significant. A possible explanation for these initial increases in ventral striatum activation is that both anticipation of an aversive and anticipation of a rewarding outcome induces dopaminergic excitation within the ventral striatum (Volman et al., 2013; Abraham et al., 2014). Nonetheless, compared to the conditioning phase, ventral striatum activation after the entire extinction phase and following reinstatement was significantly reduced for the counterconditioned but not the non-counterconditioned stimulus. Because the current paradigm did not seem to induce extinction of the reward CR to the non-counterconditioned stimulus, the psychological mechanism underlying the attenuation of the ventral striatal response remains unclear. The reduction of its response after reinstatement due to competitive positive and negative associations is consistent with the ventral striatal role in value coding. However, the initial non-selective increase during extinction appears more consistent with salience signaling. Yet another interpretation is that counterconditioning attenuates the reinstatement of reward, though the ventral striatal response to the non-counterconditioned stimulus did not decrease over time. Future studies should aim to disentangle these interpretations, possibly by altering stimulus value by manipulating the rewarding and aversive reinforcers, as well as the number of extinction trials to obtain full extinction prior to reinstatement. Regardless of these possible interpretations, this study demonstrates that reward signaling in the ventral striatum can be attenuated using aversive counterconditioning.

An important limitation of this study is that a relatively large proportion of the recruited subjects (34.6%) remained unaware of the CS–US contingency and was therefore excluded from the analysis. This large percentage of subjects unaware of the CS–US contingency could be explained by the relatively short counterconditioning phase (each CS and the US was only presented three times) and future studies may consider using a longer counterconditioning phase. Nevertheless, even this short counterconditioning phase showed to be effective in two-thirds of the participants. Impaired CS–US learning might even form a risk for impaired decision making, risky behavior or even addiction, mediated by an abnormal interaction between aversive and appetitive processes, which could be explored in future studies. A second limitation is that our paradigm did not include a behavioral measure of reward reinstatement as all subjects were instructed to keep responding to the target, even though the trials were not rewarded. As such, the only behavioral measure of reward reinstatement is a difference in valence rating for the counterconditioned and non-counterconditioned stimulus. Future studies should therefore include a more detailed measure of reward reinstatement in order to investigate the behavioral effect of counterconditioning on reward reinstatement. Third, it could be argued that the changes in ventral striatal activation or connectivity are related more to motor preparation instead of reward motivation. Similar to other studies (Knutson et al., 2000), we have however, instructed the participants to respond to the target, irrespective of whether or not they thought they could gain a reward in the trial. Doing so we have aimed to minimalize the possible confounding effect of motor preparation on ventral striatal activation and connectivity, although a motor-preparation effect cannot be fully ruled out.

## Conclusion

These results show that aversive counterconditioning prevents reward-signaling of the ventral striatum following reinstatement in healthy volunteers, thereby providing important evidence of how aversive counterconditioning could reduce appetitive CRs.

## Author Contributions

Data were obtained by AK and RS. AK, RS, and GvW analyzed the data. The first draft was prepared by AK. RS, GvW, JH, LR, and WvdB actively participated in writing and revising the manuscript for publication.

## Conflict of Interest Statement

The authors declare that the research was conducted in the absence of any commercial or financial relationships that could be construed as a potential conflict of interest.

## Footnotes

1 http://www.fil.ion.ucl.ac.uk/spm

## Abbreviations

CR: conditioned response
CS: conditioned stimulus
CS+_cc_: counterconditioned stimulus
CS+_nc_: non-counterconditioned stimulus
US: unconditioned stimulus
